# Identification and expression pattern analysis of the *OsSnRK2* gene family in rice

**DOI:** 10.3389/fpls.2022.1088281

**Published:** 2022-12-13

**Authors:** Tongyuan Yu, Qiwen Cen, Lihua Kang, Wangshu Mou, Xiaoqin Zhang, Yunxia Fang, Xian Zhang, Quanxiang Tian, Dawei Xue

**Affiliations:** College of Life and Environmental Sciences, Hangzhou Normal University, Hangzhou, China

**Keywords:** rice, *OsSnRK2*, genome−wide, abiotic stress, expression patterns

## Abstract

Sucrose non-fermenting-1-related protein kinase 2 (SnRK2) is a class of plant-specific serine/threonine (Ser/Thr) protein kinase that plays an important role in rice stress tolerance, growth and development. However, systematic bioinformatics and expression pattern analysis have not been reported. In the current study, ten *OsSnRK2* genes were identified in the rice genome and located on 7 chromosomes, which can be classified into three subfamilies (I, II, and III). Many *cis*-regulatory elements were identified in the promoter region of *OsSnRK2* genes, including hormone response elements, defense and stress responsive elements, indicating that the *OsSnRK2* family may play a crucial role in response to hormonal and abiotic stress. Quantitative tissue analysis showed that *OsSnRK2* genes expressed in all tissues of rice, but the expression abundance varied from different tissues and showed varietal variability. In addition, expression pattern of *OsSnRK2* were analyzed under abiotic stress (salt, drought, salt and drought) and showed obvious difference in diverse abiotic stress. In general, these results provide useful information for understanding the *OsSnRK2* gene family and analyzing its functions in rice in response to ABA, salt and drought stress, especially salt-drought combined stress.

## Introduction

Plants are exposed to various biotic and abiotic stresses in the natural environment that inhibit normal growth and development. During the evolution, plants have developed complicated and sophisticated mechanisms to cope with various adversity stresses. Protein kinase phosphorylation plays an important role in the signaling pathway, plant growth and development, hormone response and anti-stress by modifying the activity of translated proteins, regulatory enzymes, and various functional proteins (Umezawa et al., 2013; Mao et al., 2020; Sharif et al., 2020; Maszkowska et al., 2021).

Sucrose non-fermenting-1-related protein kinase (SnRK) is a class of serine/threonine (Ser/Thr) protein kinases and widely observed in plants. According to the conservation of the active domain of the kinase, it can be classified into three subfamilies: SnRK1, SnRK2, and SnRK3 (Hrabak et al., 2003). The SnRK2 gene family is a family of plant-specific protein kinases and involved in stress responsive processes. Recent studies have shown that members of the SnRK2 gene family play an important role in stress response by phosphorylation modifications and regulating protein activity and gene expression, thereby participating in the regulation of osmotic stress, stomatal movement through signal transduction (Riichiro et al., 2002; Taishi et al., 2004; Cheng et al., 2017; Fatima et al., 2020; Xu et al., 2020).

As one of important phytohormones, abscisic acid (ABA) is not only involved in plant growth and development but also plays an essential role in plant response to biotic and abiotic stresses (Mahadi et al., 2022). ABA signaling pathway is consisted of ABA receptor, protein phosphatase type 2C and SNF1-related protein kinase (SnRK2) (Li et al., 2000; Taishi et al., 2004; Zhang et al., 2020). In the absence of ABA, PP2C interacts with OsSnRK2 and inhibits its kinase activity, which shuts down the ABA signaling pathway; in the presence of ABA, the PYR/PYL/RCAR receptor protein binds to the hormone to form a complex, which in turn binds to PP2C, relieving its inhibition on SnRK2 and activating the ABA signaling pathway. Therefore, SnRK2 is a key factor with positive regulation of ABA signaling, and the activity of SnRK2 kinase is critical for ABA signaling on/off and abiotic stress response in plants (Fujii et al., 2007; Cheng et al., 2017).

Salt stress and drought stress have become major factors in inhibiting plant growth, development and crop yield. The main risks that the plants suffered from high concentrations of salt in the soil include osmotic stress, ion poisoning, water losing, wilting and metabolic disruption (Kawa et al., 2020; Zelm et al., 2020). In addition, salt stress causes oxidative stress, which destabilizes cell membranes (Munns and Tester, 2008). Drought stress also causes osmotic stress, which leads to the reduction of plant height, leaf area and stomatal conductance, as well as affecting plant root development (Khatun et al., 2021). Numerous studies have reported that SnRK2 is involved in plant responses to salt stress and drought stress. Under salt stress, overexpressing *OsSAPK4* reduces the accumulation of Na^+^ and Cl^-^ in rice leaves than the wild type and promote germination, growth and development (Diedhiou et al., 2008). Among the 11 SnRK2 members in maize, the expression of *ZmSnRK2.3* and *ZmSnRK2.6* were activated and significantly up-regulated by salt stress (Huai et al., 2008). In recent years, it was found that expression of most *VrSnRK2* genes was induced by drought stress, suggesting their potential function in drought stress response; with *VrSnRK2.6c* being most significantly (12-fold) induced by drought stress (Fatima et al., 2020). Overexpression of *TaSnRK2.9* in tobacco enhanced the tolerance of tobacco seedlings and mature plants to drought and salt stress, and increased tobacco survival, seed germination, and root length (Feng et al., 2019). Then, it has been shown that overexpressing *MpSnRK2.10* in *Malus prunifolia* exhibit enhanced drought tolerance in phenotypic appearance associated with drought stress damage, i.e. most of the leaves of transgenic plants appear healthy upon rehydration after drought, while the leaves of wild-type plants show extensive necrosis. Moreover, transgenic plants were observed with less accumulation of ROS and MDA than WT under drought stress (Shao et al., 2018). It has also been confirmed that transgenic plants overexpressing *OsSGT1* showed less tolerant to salt stress and that *OsSAPK9* can act as a positive regulator for salt stress response and disease resistance by interacting with *OsSGT1* (Zhang et al., 2019). All the above results implied that OsSnRK2 family may plays a crucial role in regulating plant response to salt stress and drought stress.

To better understand the OsSnRK2 family members and their responses to abiotic stress, bioinformatics analysis was performed in this study including the physicochemical properties, conserved sequences, gene structures, *cis*-acting elements, expression profiles, and phylogeny of *OsSnRK2*. In addition, the expression pattern of *OsSnRK2* in different tissues and under different stresses (ABA, salt, drought and salt-drought double) were investigated by qRT-PCR. The results will help to enrich the understanding of the *OsSnRK2* gene family and provide a theoretical basis for further studying the function of OsSnRK2 in abiotic stress response of rice.

## Materials and methods

### Gene identification and chromosome localization

The candidate OsSnRK2 protein sequences were searched for structural domains using the online website SMART: Main page (http://smart.embl-heidelberg.de/) to confirm the presence of the conserved SnRK family domain S_TKc. Based on the OsSnRK2 protein sequences reported in the literature (Yuhko et al., 2004), after collecting the gene information through the online website RiceDate (https://www.ricedata.cn/gene/), TBtools (Chen et al., 2020) was used to chromosome localization maps.

### Gene structure and conserved motifs analysis

The protein-coding regions (CDS) and DNA sequences of *OsSnRK2* were downloaded in FASTA format from the Whole Genome Data Ensembl website (http://plants.ensembl.org/index.html). The distribution of introns and exons and non-coding regions of the genes were mapped using TBtools. Conserved motifs of the proteins were analyzed online *via* the MEME website (http://meme-suite.org/) with Motif set to 12 and parameters set to default values, and conserved motifs were mapped using TBtools.

### Protein sequence alignment, phylogenetic tree, gene duplication and synteny analysis

Sequence alignment and output of the conserved structural domain of OsSnRK2 proteins were completed by MEGA7 software and geneDoc software and construction of phylogenetic relationships of the OsSnRK2 family, *Arabidopsis thaliana* and *Hordeum vulgare* was completed using MEGA7 software. The segmental repeat events of the *OsSnRK2* genes were analyzed using TBtools and presented in the form of Circos plots. Also, TBtools was used to do synteny analysis of *SnRK2* genes in genomes.

### Protein physicochemical properties, subcellular localization, secondary and 3D structure prediction

Protein physicochemical property analysis *via* ExPASy (https://web.expasy.org/protparam/) online, subcellular localization and nuclear localization were analysis by WoLF (https://wolfpsort.hgc.jp/) and NucPred (https://nucpred.bioinfo.se/uncared/) online respectively. Secondary structure predictive of protein was performed by the SOPMA website (https://npsa-prabi.ibcp.fr/cgi-bin/npsa_automat.pl?page=npsa_sopma.html). Protein 3D structure prediction for OsSnRK2 gene families using the UniProt website (https://www.uniprot.org/).

### Protein interaction and *cis*-acting elements in the promoter region of the *OsSnRK2* gene analysis

The STRING (https://cn.string-db.org/) website was used to predict the putative protein-protein interaction networks with *Oryza sativa* SnRK2 proteins using default settings. Optimising protein-protein interaction networks with Cytoscape (Doncheva et al., 2019).

The sequence of the 2000 bp region upstream of the start codon of the rice OsSnRK2 family gene was downloaded from the whole genome data Ensembl website (http://plants.ensembl.org/index.html) as the promoter sequence. Prediction analysis of *cis*-acting elements in the promoter region of the gene was performed through the PlantCare website (http://bioinformatics.psb.ugent.be/webtools/plantcare/html/) and promoter *cis*-acting elements were mapped by TBtools.

### Plant growth and stress treatments

The rice material used in this study was japonica rice Nipponbare. The International Rice Research Institute (IRRI) nutrient solution (the pH adjusted to 5.0-5.5 with KOH) was used as the hydroponic medium and changed every 3 days. The seedlings were grown in an artificial climate chamber under light conditions of 14 h at 28°C and 10 h in darkness at 24°C and 70% humidity.

ABA treatment: Rice seedlings of two leaves stage were transferred to a hydroponic medium containing 0 µM, 50 µM and 100 µM ABA respectively and the shoots were taken after 24 h for gene expression analysis.

Salt, drought, and salt-drought combined stress treatments: Rice seedlings grown hydroponically to the two leaves stage were transferred to rice nutrient solutions containing 200 mM NaCl (salt treatment), 13% PEG 6000 (drought treatment) and 200 mM NaCl and 13% PEG 6000 (salt-drought double treatment) for treatment, and shoots were collected 48 h later for gene expression analysis.

### Tissue expression and expression profiling of the *OsSnRK2* gene

RNA was extracted from roots, stems, leaves, leaf sheath and panicles at the tassel stage, and then analyzed for the expression of *OsSnRK2* genes in different tissue using qRT-PCR. The primers used in this assay were listed in **Supplementary Table 1**.

The CREP database (http://crep.ncpgr.cn/crep-cgi/home.pl) was searched for full fertility expression profile data of the indica rice variety *MingChuan63* to obtain the expression of *OsSnRK2* genes in five mature organs and 24 different developmental periods, which were clustered hierarchically using TBtools.

### RNA extraction and qRT-PCR analysis

Fluorescent quantitative PCR primers were designed by Primer Premier 5 and *OsUBQ5* was selected as the internal reference gene. Total RNA was extracted from rice samples using the Total Plant RNA Kit (Tiangen Biochemical Technology Co., Ltd.) and reverse transcribed into cDNA using the AMV First Strand cDNA Synthesis Kit. PCR amplification was performed using the CFX96 Real-Time PCR Detection System (Bio-Rad, USA). The reaction conditions were pre-denaturation at 95 °C for 5 min, denaturation at 94 °C for 35 s, annealing at 63 °C for 40 s, and extension at 72 °C for 20 s. 38 amplification cycles were performed with 4 replicates for each sample and 2^-ΔΔCt^ was used for data processing.

## Results

### Gene identification and chromosome localization analysis of *OsSnRK2*

Referring to the *OsSAPK1-10* names reported in the published literature (Huai et al., 2008), bioinformatics analysis was performed after collecting gene information *via* the online website RiceData (https://www.ricedata.cn/gene/). The detailed information of genes was listed in **Tables 1**, **2**. Fox example, the amino acid lengths of the OsSnRK2 family proteins ranged from 334 to 371 aa. Meanwhile, the molecular weight is ranged from 36.99 to 41.18 kDa. The isoelectric points and the hydrophobicity values of OsSnRK2 were ranged from 4.80 to 6.06 and -0.621 to -0.208, respectively. The negative hydrophobicity values means that the OsSnRK2 proteins are all hydrophilic.

**Table 1:** *OsSnRK2* family gene information.

Gene Name	Gene ID	Accession number
*OsSAPK1*	Os03g0390200	LOC_Os03g27280
*OsSAPK2*	Os07g0622000	LOC_Os07g42940
*OsSAPK3*	Os10g0564500	LOC_Os10g41490
*OsSAPK4*	Os01g0869900	LOC_Os01g64970
*OsSAPK5*	Os04g0691100	LOC_Os04g59450
*OsSAPK6*	Os02g0551100	LOC_Os02g34600
*OsSAPK7*	Os04g0432000	LOC_Os04g35240
*OsSAPK8*	Os03g0764800	LOC_Os03g55600
*OsSAPK9*	Os12g0586100	LOC_Os12g39630
*OsSAPK10*	Os03g0610900	LOC_Os03g41460

**Table 2:** Sequence characteristics, predicted subcellular and nuclear localization of OsSnRK2.

Gene Name	Protein Number	Length(aa)	MW(kDa)	PI	GRAVY	Subcelluar Location	NLS predicts
*OsSAPK1*	Q75LR7	342	37.79	5.43	-0.208	cytoplasm	NA
*OsSAPK2*	Q0D4J7	339	37.63	5.31	-0.248	cytoskeleton	NA
*OsSAPK3*	P0C5D6	334	36.99	5.67	-0.424	cytoplasm	NA
*OsSAPK4*	Q5N942	360	40.99	6.06	-0.616	cytoskeleton	NA
*OsSAPK5*	Q7XKA8	370	41.18	5.99	-0.501	cytoskeleton	NA
*OsSAPK6*	Q6ZI44	365	40.82	5.72	-0.621	cytoskeleton	NA
*OsSAPK7*	Q7XQP4	359	40.36	5.83	-0.567	cytoskeleton	NA
*OsSAPK8*	Q7Y0B9	371	40.75	4.85	-0.303	cytoskeleton	NA
*OsSAPK9*	Q75V57	361	39.68	4.81	-0.272	cytoskeleton	NA
*OsSAPK10*	Q75H77	362	39.75	4.80	-0.281	cytoskeleton	NA

"NA" is an abbreviation for "Not Available" and indicating no predicted nuclear localization sequences.

**Figure 1:**
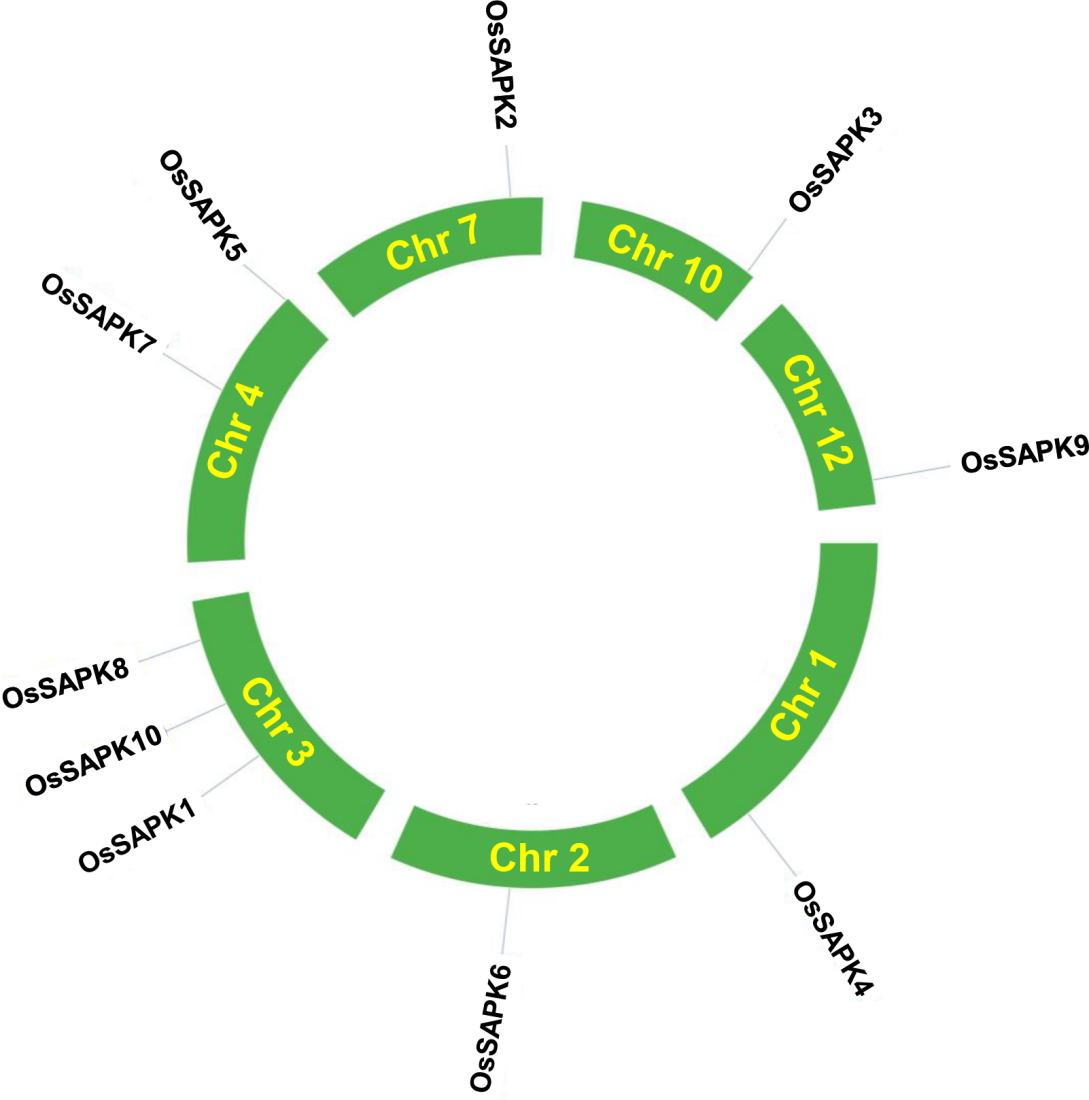
Distribution of *OsSnRK2* genes in rice chromosomes. Only show the chromosomes where located the *OsSnRK2* gene. The yellow font represents chromosome numbers and Green arc length indicate chromosome size.

Chromosome localization analysis revealed that the 10 genes of *OsSnRK2* were distributed on seven chromosomes (**Figure 1** and **Supplementary Table 2**), with *OsSAPK1*, *OsSAPK8*, and *OsSAPK10* on chromosome 3, *OsSAPK5* and *OsSAPK7* on chromosome 4, *OsSAPK4*, *OsSAPK6*, *OsSAPK2*, *OsSAPK3* and *OsSAPK9* are located on chromosomes 1, 2, 7, 10 and 12 respectively.

### Gene structure and protein motif analysis of OsSnRK2

**Figure 2:**
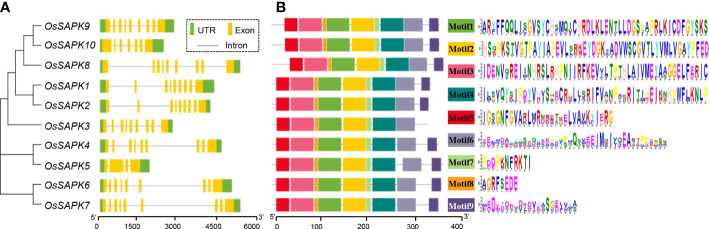
Gene structure and conserved motif analysis of the *OsSnRK2* gene. **(A)** Gene structure analysis of the *OsSnRK2*. Green boxes, yellow boxes and black lines represent UTR, Exon and Introns respectively. **(B)** Conserved motif analysis of the *OsSnRK2* gene.

The gene structure analysis of the *OsSnRK2* family members showed that the length of the family genes ranged from 1875 (*OsSAPK5*) to 5459 bp (*OsSAPK7*). *OsSAPK5* and *OsSAPK10* had four and seven exons respectively, and the rest of the *OsSnRK2* genes all contained nine exons. In addition, the last exon of most *OsSnRK2* genes was longer (**Figure 2A**).

Analysis of gene conserved motifs is useful for exploring the structural composition of proteins. We used MEME online software to analyze the number and distribution of motifs of the *OsSnRK2* gene family members, and identified a total of nine motifs, named Motif 1~Motif 9. The results showed that Motif 3 is the most conserved, and the Motif 6 ranked as the second; except for *OsSAPK3* which missing the Motif 9 conserved motif, all the other *OsSnRK2* genes contain the same number of motifs with the same order arrangement of gene structure (**Figure 2B** and **Supplementary Table 3**). The similarities and diversity of these conserved motifs may result from the evolutionary adaptation of the gene functions in *OsSnRK2* family.

### Protein sequence alignment, phylogenetic relationships, gene duplication and synteny analysis of OsSnRK2

**Figure 3:**
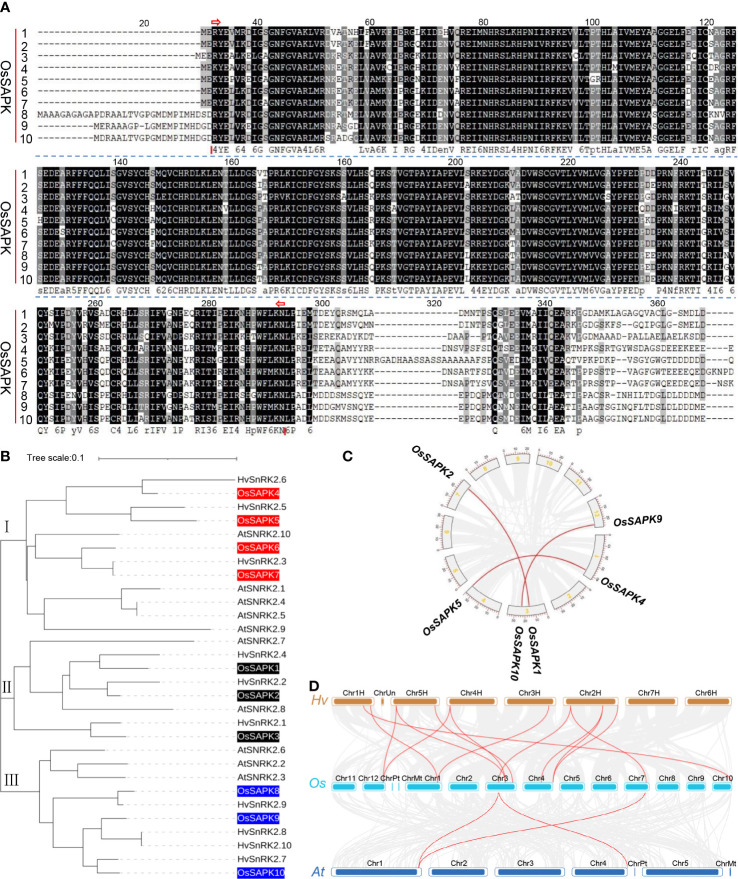
protein sequence alignment, phylogenetic relationships and gene duplication analysis of OsSnRK2. **(A)** Sequence alignment of the conserved structural domains of the *OsSnRK2* gene family, S_TKc structural domain, position 33-292, interval shown by red arrows. **(B)** Phylogenetic relationships analysis of SnRK2 in *Oryza sativa*, *Arabidopsis thaliana* and *Hordeum vulgare*. **(C)** Gene segmental duplication analysis of *OsSnRK2* in rice. Gray lines indicate all synteny blocks in the rice genome, and the red lines indicate duplicated *OsSnRK2* gene pairs. The chromosome number is indicated at the gray arc. The scale at the periphery of the chromosome represents the physical location (Kb). **(D)** Synteny analysis of *SnRK2* genes in the genomes between *Hordeum vulgare* and *Oryza sativa* or *Arabidopsis thaliana*. The gray lines show collinear blocks. The red lines indicate the syntenic gene pairs between *Hordeum vulgare* and *Oryza sativa* or *Arabidopsis thaliana*, respectively.

The alignment of amino acid composition of the OsSnRK2 protein sequences showed the presence of a highly conserved S_TKc structural domain in the OsSnRK2 proteins (**Figure 3A**), which is closely related to their highly conserved function during evolution.

To further investigate the evolutionary relationships of the OsSnRK2, phylogenetic analysis was performed with amino acid sequences of 10 OsSnRK2 and several important SnRK2 proteins from *Arabidopsis thaliana* and *Hordeum vulgare* (**Figure 3B**). The results showed that the OsSnRK2 proteins could be classified into three subfamilies according to their affinities, with *OsSAPK1*, *OsSAPK2* and *OsSAPK3* belonged to subfamily I, *OsSAPK4*, *OsSAPK5*, *OsSAPK6*, *OsSAPK7* belonged to subfamily II, and *OsSAPK8*, *OsSAPK9* and *OsSAPK10* belonged to subfamily III. The OsSnRK2 family members are closely related to the *Arabidopsis thaliana* and *Hordeum vulgare* SnRK2 family members, suggesting that they might have conserved physiological and biochemical functions.

Three segmental duplication events were identified in *OsSnRK2* family by the MCScanX function of the software TBtools (**Figure 3C**) and involving six genes of ten *OsSnRK2*. All segmental duplication events happened between different chromosomes, but all occur within the same subfamily. The above results demonstrate that a number of *OsSnRK2* genes maybe appear in the course of gene duplication, and the segmental duplication events may be responsible for the expansion of *SnRK2* genes in rice.

The colinearity of *OsSnRK2* gene pairs between *Oryza sativa*, *Hordeum vulgare* and *Arabidopsis thaliana* was compared. The result showed that 2 *OsSnRK2* genes exhibited syntenic relationship with *AtSnRK2*. However, 9 *OsSnRK2* genes showed syntenic relationship with *HvSnRK2* (**Figure 3D** and **Supplementary Table 4**), implying that these genes may play a critical role in evolution of *OsSnRK2* family.

### Subcellular localization, secondary and 3D structure prediction of OsSnRK2

The predicted subcellular localization results showed that *OsSAPK1* and *OsSAPK3* proteins were localized in the cytoplasm, the remaining OsSnRK2 proteins were localized in the cytoskeleton. The NucPred scores of the OsSnRK2 family proteins were all below 0.6, indicating no predicted nuclear localization sequences (marked with “NA” in **Table 2**).

**Figure 4:**
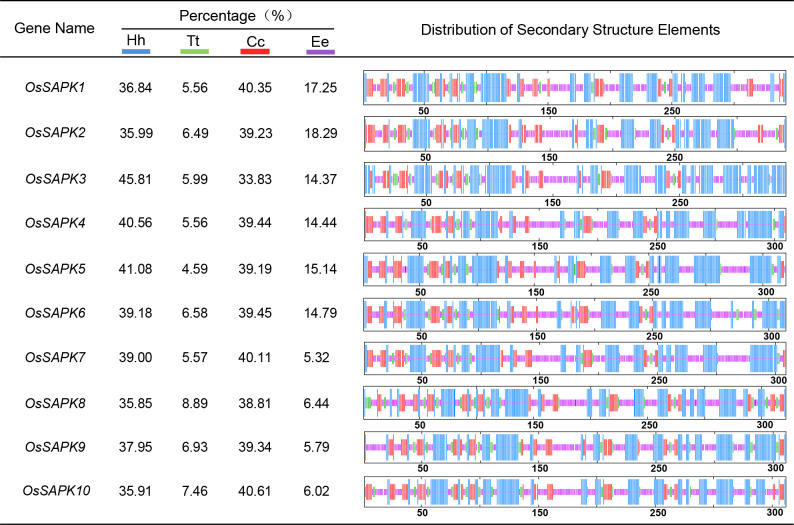
Secondary structure analysis of OsSnRK2 proteins. The blue color represents alpha helix (Hh), the green color represents extended helix (Tt), the yellow color represents random coil (Cc) and the red color represents extended strand (Ee).

**Figure 5:**
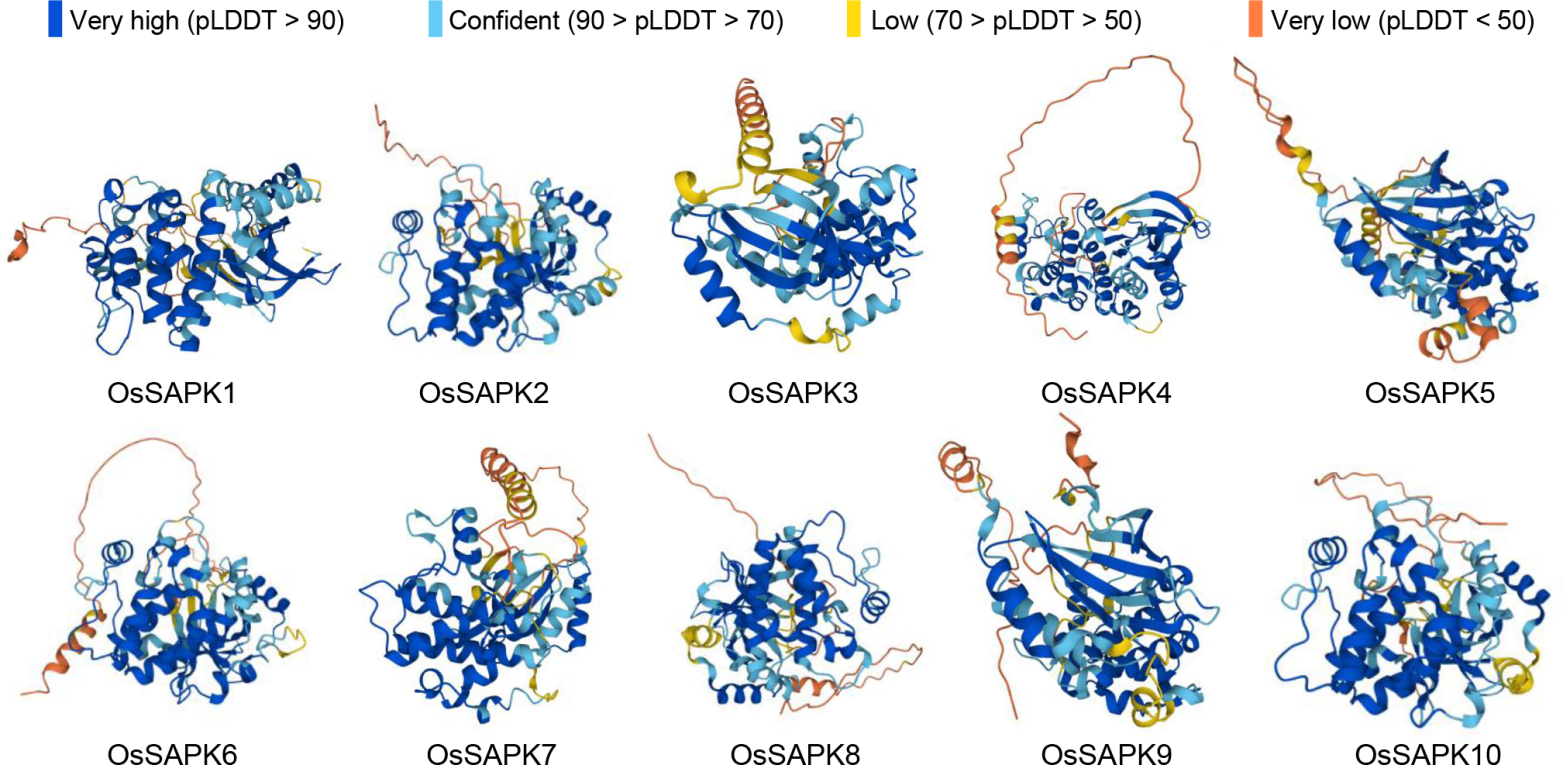
Protein 3D structure prediction model of *OsSnRK2* gene families. AlphaFold produces a per-residue confidence score (pLDDT) between 0 and 100.

Secondary structures of OsSnRK2 proteins were analyzed by the SOPMA online website (https://npsa-prabi.ibcp.fr/cgi-bin/npsa_automat.pl?page=npsa_sopma.html). All OsSnRK2 proteins were consisted of four secondary structures: alpha helix, extended helix, random coil, and extended strand (**Figure 4**), and the proportions of alpha helix > random coil > extended strand > extended helix. It is assumed that the alpha helix and random coil are the main conformations of the OsSnRK2 protein. The results of protein 3D structure prediction shows that all OsSnRK2 proteins have similar 3D structure (**Figure 5**).

### Protein-protein interaction analysis of OsSnRK2 proteins

**Figure 6:**
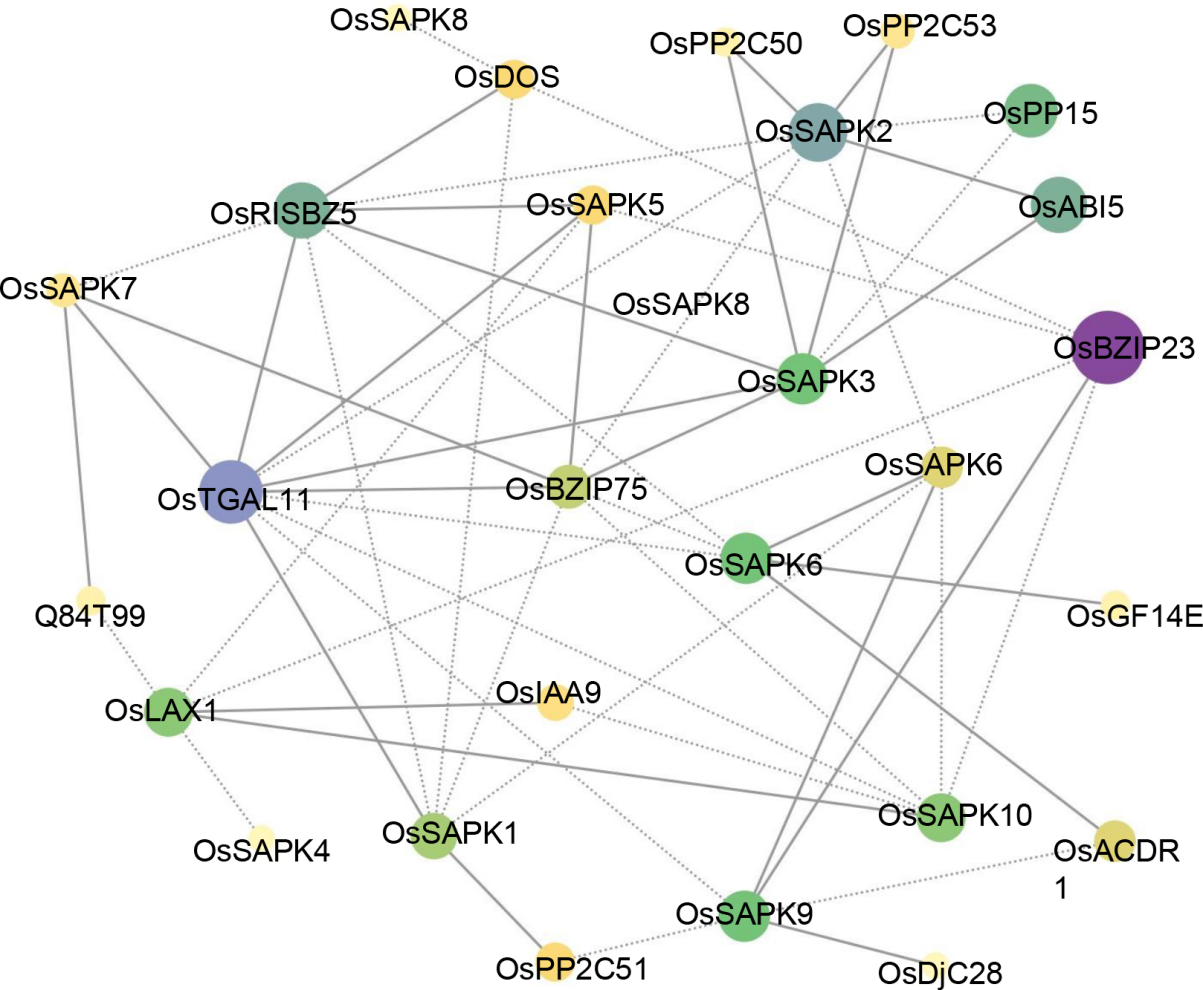
Protein-protein interaction network assembly of OsSnRK2 proteins. The area and color of the circles represent the number of interacting proteins, the larger area and darker color of the circles indicate more interacting proteins. The dashed lines represent protein interaction score ≤ 0.5, and solid lines means protein interaction score > 0.5.

To identify potential interacting proteins with the OsSnRK2 proteins, a protein - protein interaction (PPI) network was generated with the STRING database (**Figure 6**). Notably, several proteins belonging to OsBZIP and OsPP2C proteins interacted with OsSAPK1, OsSAPK2, OsSAPK3, OsSAPK5, OsSAPK6, OsSAPK7, OsSAPK9, OsSAPK10, suggested their regulatory role in ABA signaling. Furthermore, there are other potential interacting proteins for OsSnRK2 proteins. Among them, OsDOS (a CCCH-Type Zinc Finger Protein) and OsDjC28 (belongs to heat shock protein DnaJ), are both showing tight relationships with different OsSnRK2 proteins, respectively.

### *Cis*-elements analysis in *OsSnRK2* promoters

**Figure 7:**
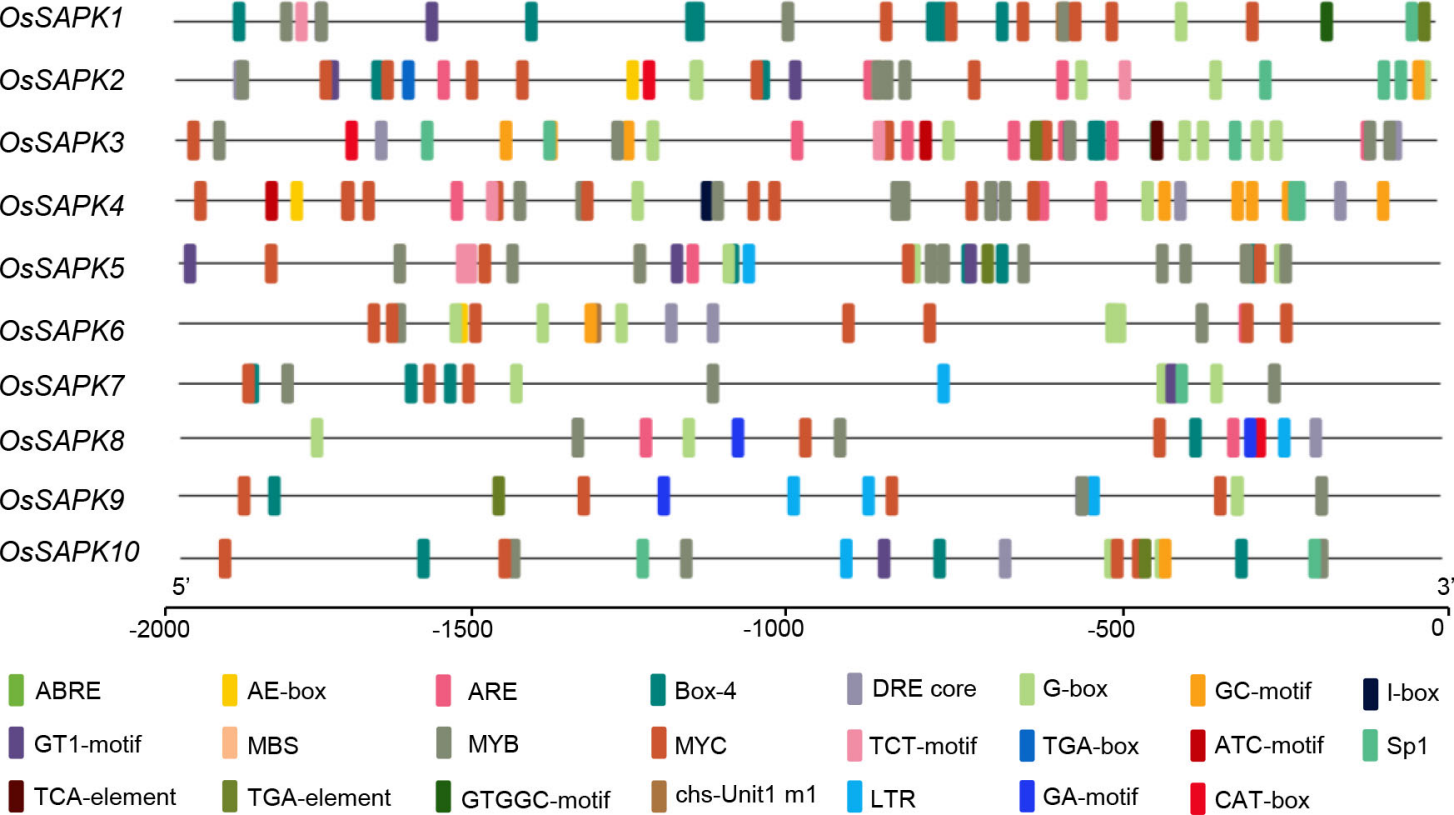
Predicted *cis*-acting elements in *OsSnRK2* promoter. Promoter sequences (−2 Kb) of 10 *OsSnRK2* were analyzed by PlantCARE. Different *cis*-elements are represented by different colors.

To explore the underlying function of the *OsSnRK2* genes, Plant-CARE was adopted for the analysis of *cis*-elements in their promoter region. The sequence of 2000 bp upstream of the *OsSnRK2* gene start codon was downloaded from the whole genome data Ensembl website and submitted for the *cis*-elements assay. The promoter sequences of the *OsSnRK2* gene contained a number of light-responsive *cis*-acting elements (e.g. AE-box, Box4, G-Box, GT1-motif, sp1), ABA-responsive *cis*-acting elements (ABRE), stress-responsive *cis*-acting elements (DRE, LTR, MYB, MYC, TC-rich repeats), salicylic acid response-related elements (TCA-element), MeJA-related elements (CGTCA-motif, TGACG-motif) and meristematic tissue expression elements (CAT-box) (**Figure 7** and **Supplementary Table 5**). This indicates that *OsSnRK2* genes may be by exogenous factors such as light, hormones, stress and involved in the stress response.

### Tissue expression and expression profile analysis of *OsSnRK2*

**Figure 8:**
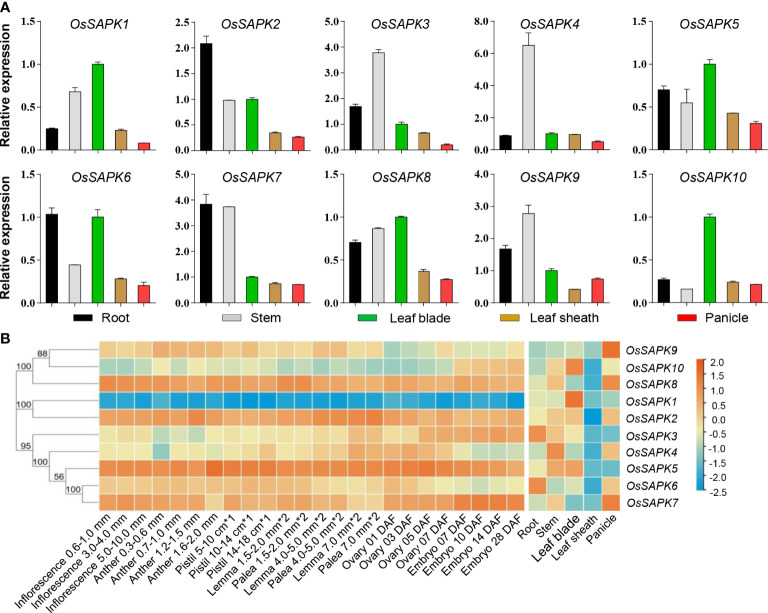
Expression analysis of the *OsSnRK2* in different tissues. **(A)** Expression analysis of *OsSnRK2* in root, stem, leaf blade, leaf sheath and panicle by qRT-PCR. *OsUBQ5* was used for normalization. The data represent means ± standard deviation (n = 3). **(B)** Expression profiles of *OsSnRK2* in an indica rice variety Minghui 63 obtained from CREP database. The color scale represents relative expression levels from low (blue) to high (red).

The expression patterns of the *OsSnRK2* gene were investigated in different tissues (root, stem, leaf, sheath and panicle) of rice (Nipponbare) (**Figure 8A**). It was found that *OsSAPK2* and *OsSAPK7* were most abundantly expressed in the root and expressed at the lowest level in the panicle; *OsSPK3*, *OsSAPKA4*, and *OsSAPK9* were the most highly expressed in the stem, *OsSAPK3*, *OsSAPK4* were the least expressed in panicle and *OsSAPK9* was the least expressed in leaf sheath; *OsSAPK1*, *OsSAPK5*, *OsSAPK8*, and *OsSAPK10* were the most highly expressed in leaves, *OsSAPK1*, *OsSAPK5* and *OsSAPK8* were the least expressed in panicle and *OsSAPK10* was the least expressed in stems. These results indicated that the expression pattern of the ten *OsSnRK2* genes were tissue-specific, suggesting that *OsSnRK2* might play distinct roles in different tissue in growth and stress response of rice.

The expression file of the *OsSnRK2* genes of Indica rice *Mingchu 63* obtained from the CREP database (https://ricexpro.dna.affrc.go.jp/data-set.html) was hierarchically clustered in root, stem, leaf blade, leaf sheath, and panicle (**Figure 8B** and **Supplementary Table 6**), and the results showed that the expression level of *OsSAPK1* and *OsSAPK10* were highest in leaves, *OsSAPK2* was highest in stems and *OsSAPK8* was lowest in panicles, and these were consistent with the expression pattern of the *OsSnRK2* genes that we showed in the japonica rice variety Nipponbare. However, the expression pattern of *OsSAPK7* and *OsSAPK9* obtained from the CREP database in *Mingchu* 63 differed dramatically from that in Nipponbare, with *OsSAPK7* and *OsSAPK9* in *Mingchu* 63 having the highest expression in the panicle. Taken together, the results indicate that there is some variability in the tissue expression of *OsSnRK2* among different rice varieties.

Meanwhile, the expression of the *OsSnRK2* family genes of Indica rice *Mingchu 63* obtained from the CREP database was hierarchically clustered at 24 different developmental stages (**Figure 8B** and **Supplementary Table 6**). On the basis of the expression patterns of the *OsSnRK2* genes in the reproductive period, they could be classified into three groups. Group 1 had low expression in all periods and contained *OsSAPK1*. Group 2 include *OsSAPK2*, *OsSAPK5*, *OsSAPK7*, and *OsSAPK8*, and they were expressed at relatively high level in most periods. The rest of *OsSnRK2* genes were belong to the Group 3 and had moderate expression during all the periods. These results implied that *OsSnRK2* showed different expression level in different developmental periods.

### Expression patterns of *OsSnRK2* under ABA and abiotic stress

**Figure 9:**
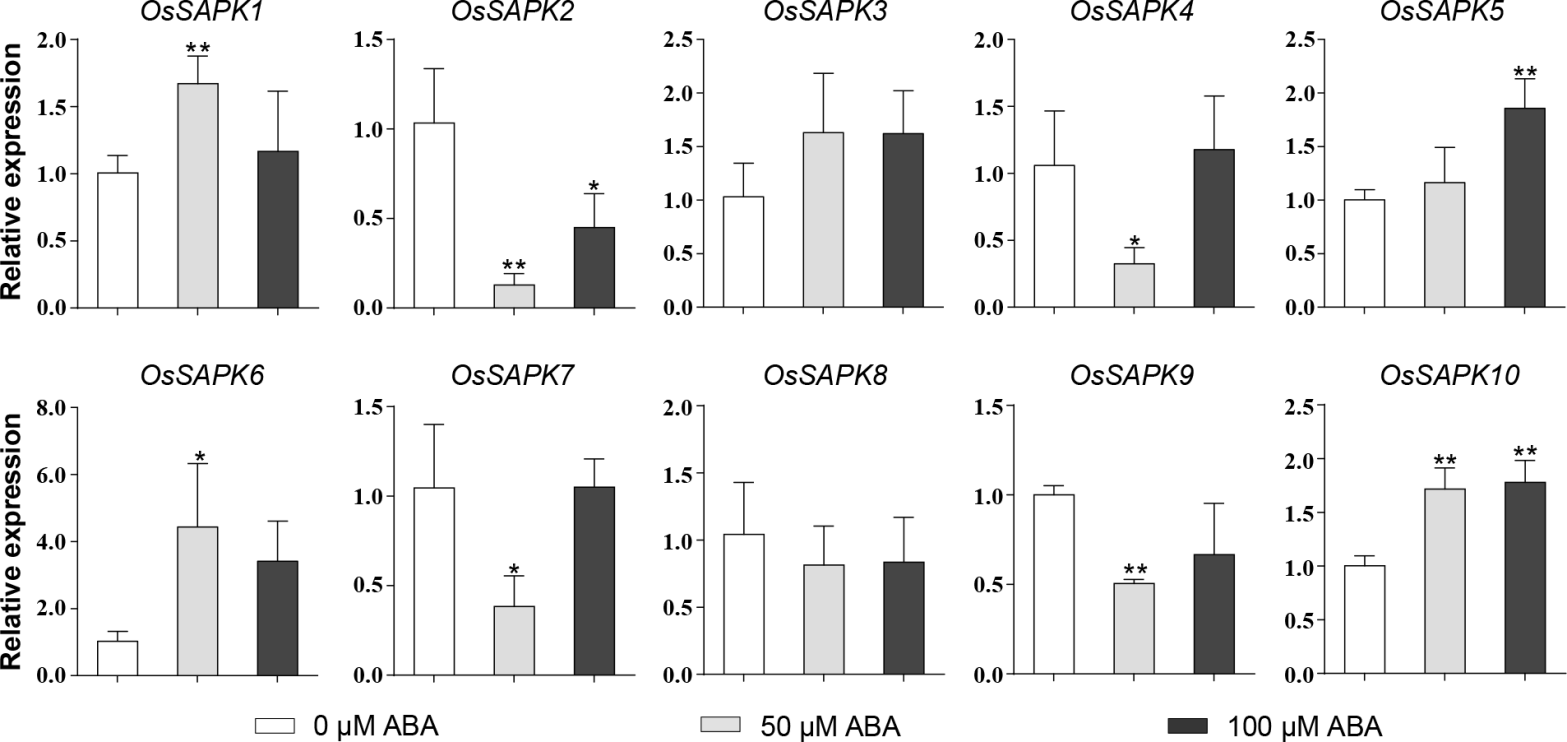
Expression analysis of *OsSnRK2* under different ABA concentration treatments. *OsUBQ5* was used for normalization. Total RNA was extracted from shoots of 14-day-old rice seedlings that were treatment or not. The data bars indicated Mean ± SD of three replicates. Asterisks represents significant differences from 0 µM ABA treatment (*p < 0.05; **p < 0.01) by Student’s *t* test.

To investigate the response of the *OsSnRK2* genes to ABA, the expression level of *OsSnRK2* genes in rice seedlings after 24 h treatment with different concentrations of ABA (0 µM, 50 µM, 100 µM) was analyzed (**Figure 9**). The results showed that the expression of *OsSAPK3* and *OsSAPK8* remained unchanged under different concentrations of ABA applications; the expression trends of *OsSAPK2*, *OsSAPK4*, *OsSAPK7*, and *OsSAPK9* were significantly lower than that of the control with 50 µM ABA treatment. The expression of *OsSAPK2*, *OsSAPK4*, *OsSAPK7*, and *OsSAPK9* was 0.002-fold, 0.31-fold, 0.37-fold, and 0.51-fold lower than the control with 50 µM ABA treatment, respectively; the expression of *OsSAPK1* and *OsSAPK6* tended to increase and then decrease with increasing concentration of ABA treatment. The expression levels of *OsSAPK1* and *OsSAPK6* in 50 µM ABA treatment were 1.66 and 4.32 times higher than the control group, respectively. The expression levels of *OsSAPK5* and *OsSAPK10* were enhanced as the ABA concentration increased, and the levels of *OsSAPK5* and *OsSAPK10* in 100 µM ABA treatment were 1.85 and 1.77 times higher than the control group, respectively.

**Figure 10:**
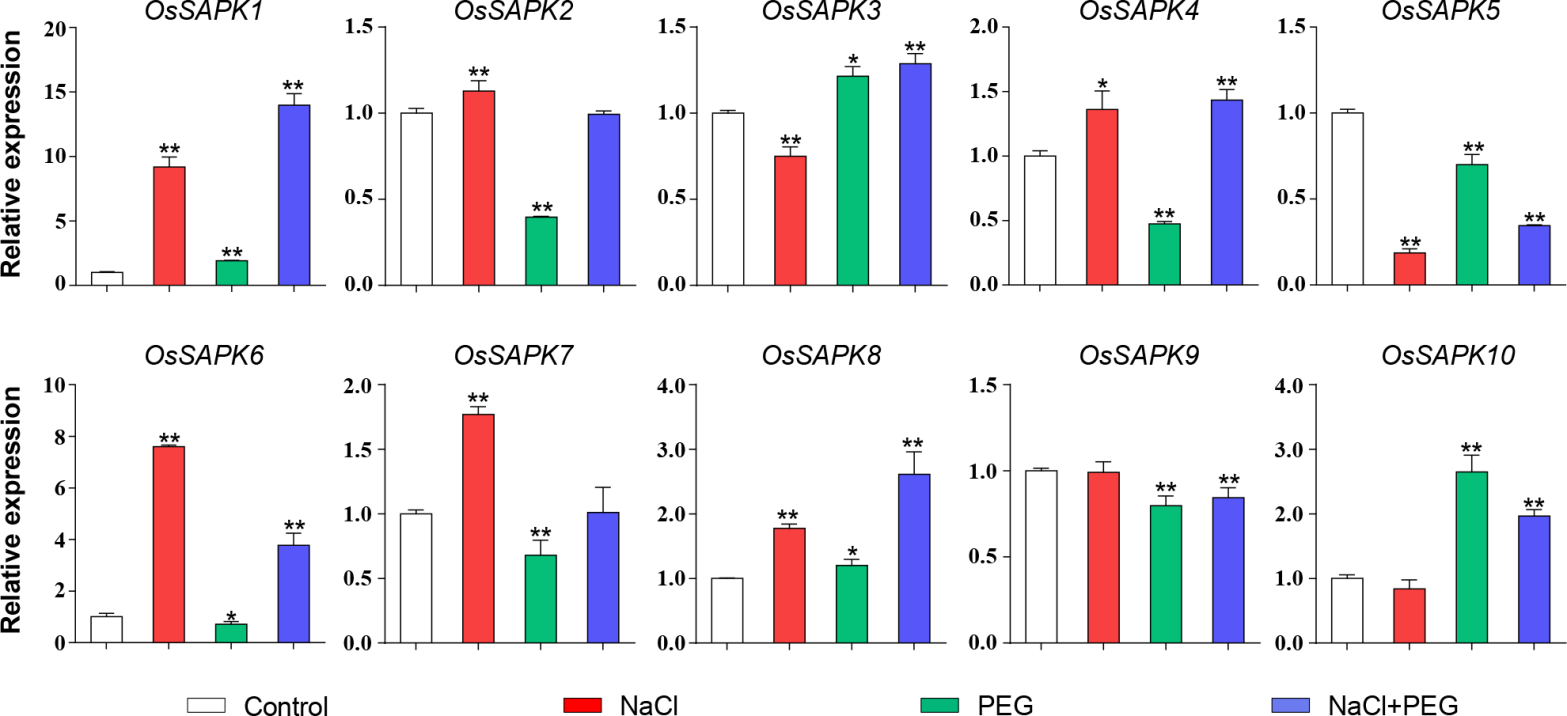
Expression analysis of *OsSnRK2* under salt, drought and salt-drought double stress. *OsUBQ5* was used for normalization. Total RNA was extracted from shoots of 14-day-old rice seedlings that were treatment or not. The data bars indicated Mean ± SD of three replicates. Asterisks represents significant differences from Control (*p < 0.05; **p < 0.01) by Student’s t test.

To investigate the gene expression of *OsSnRK2* gene under salt and drought stress, the transcription level of the *OsSnRK2* gene in rice seedlings under three different treatments of 200 mM NaCl (salt treatment), 13% PEG 6000 (drought treatment) and 200 mM NaCl combined with 13% PEG 6000 (salt-drought double treatment) was compared using qRT-PCR (**Figure 10**). Under salt stress, the expression level of *OsSAPK1*, *OsSAPK2*, *OsSAPK4*, *OsSAPK6*, *OsSAPK7* and *OsSAPK8* were dramatically increased. In contrast, *OsSAPK3* and *OsSAPK5* were significantly decreased. However, the expression of other *OsSnRK2*s were not altered by salt stress. Under drought stress, *OsSAPK10* was observed significantly up-regulated, which was 2.65-fold higher than the control. Some of the *OsSnRK2* genes were inhibited by drought stress, with *OsSAPK2* and *OsSAPK4* being 0.40 and 0.48 times than the control, respectively. The expression of *OsSAPK1* and *OsSAPK6* was significantly up-regulated than the other genes under the salt-drought double stress, with 13.96 and 3.75 times higher than the control, respectively. The above results demonstrate the potential important role of the *OsSnRK2* family in response to abiotic stress.

## Discussion

### Detailed characterization and evolution of *SnRK2* in rice

*SnRK2* genes were found only in the plant kingdom and play a crucial role in plant growth, stress response, and have been identified in a variety of plants, such as *Arabidopsis thaliana*, *oat*, *Hevea brasiliensis* (Cheng et al., 2017; Shao et al., 2018; Wang et al., 2019), etc. Researchers has also identified *OsSnRK2* in rice, but not systematically. In the current study, ten *OsSnRK*2 gene (named *OsSAPK1*-*OsSAPK9*) sequences were obtained from website RiceDate for detailed bioinformatics analysis. Ten *OsSnRK2* genes were unevenly distributed on seven chromosomes (**Figure 1**). The same subfamily genes often share similar intron-exon organization and motif (Zhang et al., 2022), For example, most *SnRK2* genes have 9 exons in *Nicotiana tabacum* (Li et al., 2022). In this study, we found that majority of *OsSnRK2* genes have 9 exons and the same motif arranged in the same order (**Figure 2**). The similarity shown above implied the functional redundancy among the members of *OsSnRK2* gene family. However, there were differences in the structure of individual *OsSnRK2* compared with genes of the same subclade. For example, the number of exons of *OsSAPK5* is significantly less than other members in the same subfamily, which only has 5 exons, and *OsSAPK3* has 8 motif, but others member have 9 motif. It is speculated that *OsSnRK2* members may have new functions by increased or decreased introns in the process of evolution to adapt to environmental changes.

Multiple sequence alignment of OsSnRK2 proteins showed that the N-terminus of this family was highly conserved protein kinase catalytic domain (S_TKc structural) and contained similar motif composition, which has also been reported in pepper (*Capsicum annuum* L.) (Wu et al., 2020; **Figure 3**). In the analysis of gene duplication and collinearity of *OsSnRK2*, we identified three pairs of segmental duplication events in ten genes, which speculated that segmental duplication may be the main way of *SnRK2* gene expansion in rice. A pair of duplication events, *OsSAPK1* and *OsSAPK2*, have been confirmed to act synergistically and become positive regulators of salt tolerance in rice (Lou et al., 2018). Moreover, the more syntenic gene pairs between *Hordeum vulgare* and *Oryza sativa* than between *Oryza sativa* and *Arabidopsis thaliana*, illustrating the greater affinity between *Hordeum vulgare* and *Oryza sativa*, which was further demonstrated by the results of phylogenetic relationships analysis (**Figure 3**).

### *OsSnRK2* genes play important roles in ABA pathway

The results of protein-protein interaction analysis show that the OsSnRK2 proteins are closely related to the OsBZIP and OsPP2C proteins (**Figure 6**). Among the OsBZIP proteins, OsBZIP23 and OsBZIP46 are positive regulators in ABA-mediated drought resistance and are involved in ABA signaling (Yoshida et al., 2010; Guo et al., 2013; Vanitha et al., 2022). In addition, among the OsPP2C protein family, there are key players in ABA signal transduction, including OsPP2C50 and OsPP2C53, which act by negatively regulating ABA responses (Rodriguez et al., 1998; Merlot et al., 2001; Ma et al., 2009; Li et al., 2015; Zhao et al., 2021). These proteins have been shown to interact with some of the OsSnRK2 family proteins, suggesting that the OsSnRK2 family proteins are widely involved in the ABA signaling pathway.

*Cis*-acting elements are important switches in the regulation of gene transcription. Analysis of *cis*-acting elements of *OsSnRK2* promoter showed that there were enriched in *cis*-elements for the responses to ABA, such as ABRE, AER, etc. (**Figure 7**). This results are similar to those in pepper (Wu et al., 2020), Brassica napus (Yoo et al., 2016), *Ammopiptanthus nanus* (Tang et al., 2021). Further analysis revealed that 90% of *OsSnRK2* genes had ABRE, an ABA-related regulatory element (Kazuo and Kazuko, 2013), suggesting that the potential involvement of *OsSnRK2* family genes in ABA response. For example, mutations in ABA-activated SNF1-associated protein kinase 2 (SnRK2s) – SRK2D/SnRK2.2, SRK2E/SnRK2.6/OST1 and SRK2I/SnRK2.3 in *Arabidopsis thaliana* cause up-regulation of ABA repressor gene expression and down-regulation of ABA activator gene expression, resulting in severe growth defects during seed development, such as loss of dormancy function (Kazuo et al., 2009).

Protein interaction and *cis*-acting elements analysis of OsSnRK2 indicate that this family likely involved in ABA signaling transduction pathway, which was further confirmed by the expression analysis of *OsSnRK2* gene under ABA treatment. Most *OsSnRK2* genes expression were significantly altered by exogenous ABA, some were up-regulated (*OsSAPK1*, *6*, *5*, *10*) and some were down-regulated (OsSAPK*2*, *4*, *7*, *9*), indicating that *OsSnRK2* genes play a dual role in ABA response and plant growth (**Figure 9**). Mahadi et al. demonstrate this point and proposed that *OsSnRK2* can promote plant growth under normal conditions and while inhibiting plant growth in the absence of ABA (Mahadi et al., 2022).

### *OsSnRK2* genes play important roles in salt and drought stress responses

In the natural environment, plants are subjected to various abiotic stresses, such as drought, salinity low temperature, etc. As protein kinases, OsSnRK2 can phosphorylate or dephosphorylate the interacting proteins and involved in signal transduction, which is essential for plants to sense and adapt to various stress (Lou et al., 2017; Wang J et al., 2020; Wang Y et al., 2020; Li et al., 2021; Jia et al., 2022). In current study, we identified kinds of potential interacting factor for OsSnRK2 proteins including heat shock protein DnaJ and bZIP transcription factors that play a crucial role in salt and drought stress, and indicating OsSnRK2 involved in stress response (**Figure 6**). In addition, a large number of *cis*-acting elements were found in the *SnRK2* genes promoters, which is important in response to different hormones and abiotic stresses in wheat, potato, cotton (Zhang et al., 2016; Bai et al., 2017; Liu et al., 2017). Similar results were also verified in rice. The promoters of almost all *OsSnRK2* contain drought and salt stress response *cis*-acting elements, such as ABRE, G-box, and DRE, indicated that *OsSnRK2* family play a crucial role in the process of rice adaptive stress.

Previous studies have demonstrated that *SnRK2* genes are involved in a various of abiotic stresses responses (Ding et al., 2015; Soma et al., 2017; Tan et al., 2018). AtSnRK2.4 and AtSnRK2.10 are rapidly and transiently activated and regulate ROS homeostasis involved salt stress in *Arabidopsis* (Szymańska et al., 2019). The expression of *TaSnRK2* genes was induced by drought and salt, and overexpression of *TaSnRK2.4* in *Arabidopsis* significantly increased salt tolerance (Zhang et al., 2016). Under salt and PEG treatment, five *GhSnRK2* genes expression were found notably upregulated in cotton (*Gossypium hirsutum*) (Liu et al., 2017). In *Populus trichocarpa*, heterologously overexpression of *PtSnRK2.5* and *PtSnRK2.7* genes could enhance *Arabidopsis* salt stress tolerance (Song et al., 2016). In rice, several *SnRK2* genes have also been identified to be involved in stress responses. For example, overexpression of *OsSAPK4*, *OsSAPK6* or *OsSAPK7* in rice could increase salt tolerance (Diedhiou et al., 2008; Nam et al., 2012; Zeng et al., 2021), and overexpression *OsSAPK2* could improve grain yield by regulating nitrogen utilization under reproductive drought stress (RDS) (Lou et al., 2020). However, there is no systematic gene expression analysis under abiotic stress, especially under salt-drought combined stress, though they can coexist in the agroecosystem. In our results, almost all *OsSnRK2* members responded to salt and drought treatment simultaneously, except *OsSAPK9* and *OsSAPK10* which only responded to drought stress. Some *OsSnRK2*s showed opposite responses to salt and drought, but some are the same. For example, *OsSAPK1* and *OsSAPK8* expression were up-regulated by salt and drought simultaneously, and *OsSAPK2*, *OsSAPK4*, *OsSAPK6* and *OsSAPK7* were up-regulated by salt and down-regulated by drought stress (**Figure 10**). Under salt and drought stress, most *OsSnRK2* genes, including *OsSAPK1*, *OsSAPK3*, *OsSAPK4*, *OsSAPK6*, *OsSAPK8* and *OsSAPK10*, were up-regulated and two genes (*OsSAPK5*/*9*) were significantly down-regulate than control(**Figure 10**). The gene expressions of *OsSAPK1* and *OsSAPK8* under salt-drought combined stress were significantly higher than those under single stress (salt or drought). In addition, *OsSAPK1* and *OsSAPK8* were highly induced by salt and drought simultaneously, which suggested that *OsSAPK1* and *OsSAPK8* may play an important positive regulatory role in response to salt-drought combined stress, and overexpression of *OsSAPK1* or *OsSAPK8* in rice may significantly improve the salt and drought tolerance and increase rice yield. The above results showed that *OsSnRK2* genes exhibit different expression profiles in response to salt and drought, which indicated that *OsSnRK2* genes play different role, and may be have different mechanisms in response to salt and drought stress.

## Conclusion

Here, we studied the *SnRK2* gene family in rice by bioinformatics method. The results revealed the characteristics of the *OsSnRK2* gene in terms of physicochemical properties, phylogenetic relationships, structural domain distribution, chromosomal localization, motif composition, intron-exon structure, tissue expression, etc. In addition, the tissue-specific expression and response of the *OsSnRK2* gene to ABA, salt, drought, and salt-drought double stress were analyzed using qRT-PCR. The results showed that *OsSnRK2* genes were expressed in rice roots, stems, leaves, leaf sheaths and panicles, but the expression levels varied from different tissues and varieties. The expression level of most *OsSnRK2* genes were induced by ABA, salt, drought, and salt-drought double stress, indicating that *OsSnRK2* genes may be extensively involved in stress response. In summary, this study adopted bioinformatics way and qRT-PCR to unveil the physicochemical properties, tissue expression patterns, and responses of the *OsSnRK2* gene to different stresses, expanding the understanding of *SnRK2* gene family in rice and providing a reference for further investigation of their functions in response to ABA treatment and different abiotic stress.

## Data availability statement

The datasets presented in this study can be found in online repositories. The names of the repository/repositories and accession number(s) can be found in the article/**Supplementary Material**.

## Author contributions

DX, QT, and TY conceived and designed the research; TY, QC, LK, WM, XQZ, YF, XZ, and QT performed the experiments and data analyses. DX supervised the experiments and gave advice on laboratory work. TY and QT wrote the manuscript. DX, QT, and WM revised the manuscript. All authors read and approved the final article.
